# Predictors of response to omalizumab and relapse in chronic spontaneous Urticaria: a narrative review focusing on parameters available in routine clinical practice

**DOI:** 10.3389/falgy.2025.1688464

**Published:** 2025-10-30

**Authors:** Laura Mateu-Arrom, Xenevra Adriana Vence Nogueira, Lluis Puig, Jorge Spertino

**Affiliations:** Department of Dermatology, Hospital de la Santa Creu I Sant Pau, Institut d’Investigació Biomèdica Sant Pau (IIB SANT PAU), Universitat Autònoma de Barcelona, Barcelona, Spain

**Keywords:** chronic spontaneous urticaria, omalizumab, predictor, relapse, response, recurrence

## Abstract

Chronic spontaneous urticaria (CSU) is a heterogeneous disease with variable responses to treatment. Identifying predictors of response to omalizumab and relapse after its discontinuation is essential for optimizing management. This narrative review aims to summarize current evidence, emphasizing clinically accessible parameters to provide a practical guide for physicians in routine care settings. Response to omalizumab appears to be influenced by the underlying pathophysiological subtype of CSU. Type IIB autoimmune CSU, associated with lower total IgE levels, higher IgG anti-thyroid peroxidase levels, basopenia, eosinopenia, elevated C-reactive protein, and greater disease activity, correlates with poorer responses. Coexisting inducible urticaria is associated with the need for longer duration of omalizumab therapy. Patients with higher body mass index may be poor responders to omalizumab at licensed doses but may benefit from dose escalation. Predictors of relapse after discontinuation include high baseline disease activity, which may be related to type IIB autoimmune CSU, and longer disease duration. Achieving complete disease control prior to tapering omalizumab may also reduce the risk of recurrence. In conclusion, clinically accessible parameters can assist in predicting response to omalizumab and relapse risk. These indicators can support individualized treatment decisions and counseling in daily practice. Further research is needed to refine relapse predictors and validate strategies such as treatment optimization.

## 1 Introduction

Chronic urticaria is a condition characterized by the development of recurring wheals, angioedema, or both that last 6 weeks or longer (1). It can be classified based on the role of the presence of triggers, as inducible or spontaneous (2). Inducible urticaria is characterized by the presence of subtype-specific triggers that always lead to the development of symptoms, which never appear without the trigger (2). In contrast, spontaneous urticaria can occur without a specific trigger (2). The prevalence of chronic urticaria in adults is estimated at 0.5%–5% (3), and 20–45% of patients with acute urticaria will develop chronic urticaria (4).

Current evidence identifies dermal mast cells (MCs) as key drivers of chronic spontaneous urticaria (CSU) (5, 6). Upon activation, MCs release vasoactive mediators, including histamine, leukotrienes, platelet-activating factor, among others, which in turn promote endothelial activation, vasodilation, increased vascular permeability, and recruitment of secondary inflammatory cells (5). Although the underlying basic pathomechanisms of CSU are still largely unclear, two main immunopathogenic mechanisms have been proposed, likely affecting distinct CSU patient subgroups, although with possible overlap (5). Both mechanisms involve the extracellular *α*-subunit of the high-affinity immunoglobulin E (IgE) receptor (Fc*ε*RI) expressed on MCs and basophils (5), although other receptors on the MC surface are also responsible for MC activation (6). Type I autoimmune CSU, also referred to as autoallergic, is mediated by IgE autoantibodies that recognize autoantigens in the skin such as IL-24 or thyroid peroxidase (TPO), forming immune complexes that activate MCs (5). In contrast, type IIb autoimmune CSU is driven mainly by IgG (typically IgG1 and IgG3) targeting Fc*ε*RI or IgE (5). These antibodies cross-link Fc*ε*RI or Fc*ε*RI-bound IgE, directly activating MCs and basophils. Additionally, they can initiate complement activation, generating C5a, which further amplifies MC and basophil response (5). The distinction between type I and type IIb CSU has important clinical implications (5). Type IIb CSU, a well-defined subgroup characterized by low total IgE levels and elevated IgG anti-TPO antibodies, predominantly affects women and is characterized by late disease onset, high disease activity, frequent angioedema, and a higher prevalence of autoimmune comorbidities (5, 7).

Current European guidelines recommend second-generation H1-antihistamines at standard doses as first-line treatment, and up to fourfold increased doses as second-line therapy (2). Nevertheless, some series report that up to 60% of patients with CSU remain unresponsive to antihistamines and require third-line treatment (8–10).

Omalizumab, currently considered the third-line treatment in patients with CSU (2), is a monoclonal anti-IgE antibody that binds to free IgE, lowering free IgE levels and causing Fc*ε*RI receptors on basophils and mast cells to be downregulated (11). Additional mechanisms by which omalizumab may improve CSU symptoms include reducing mast cell releasability, restoring basophil counts and IgE receptor function, and attenuating the activity of both IgG autoantibodies targeting Fc*ε*RI/IgE and IgE autoantibodies—whether directed against known or unidentified autoantigens (11).

Omalizumab has shown excellent efficacy in controlling urticaria symptoms in patients who are refractory to antihistamines, compared to placebo (12, 13). However, approximately 35% of patients do not respond to the standard dose of 300 mg every four weeks, and notably, around 25% fail to respond even after dose escalation, requiring a switch to or the addition of cyclosporine (14).

Current guidelines recommend assessing potential biomarkers or predictors of disease course and treatment response in all patients with CSU as part of the baseline diagnostic workup (2). Although no definitive predictors have yet been validated, available markers may assist clinicians in counseling patients regarding disease severity, expected duration, and likely treatment outcomes (2).

Several clinical and laboratory markers have been associated with poor response to second-generation H1-antihistamines (sgAHs) in patients with CSU (15), such as high disease activity (16, 17), elevated C-reactive protein (CRP) levels (18, 19), the presence of chronic inducible urticaria (CIndU), particularly symptomatic dermographism and delayed pressure urticaria (16, 17) or elevation of D-dimer (20–22). Other emerging markers of sgAH nonresponse include prior corticosteroid use (23), basopenia (20), or eosinopenia (24), among others (15). Basopenia and eosinopenia are particularly associated with severe, autoimmune, and treatment-refractory forms of CSU (15, 20). Angioedema has also been observed more frequently in patients with sgAH refractory CSU (25).

Given the relevance of identifying predictive factors and the substantial number of patients with CSU requiring treatment with omalizumab, there is significant interest in understanding biomarkers associated with treatment response or relapse after omalizumab. This study is a narrative review of the literature on predictive factors of omalizumab response in patients with CSU. We specifically focus on variables that are readily available in routine clinical practice, deliberately excluding those based on experimental methods or complex laboratory techniques, with the aim of providing a practical and accessible resource for all physicians managing CSU, regardless of their clinical setting.

## 2 Predictors of non-response to omalizumab

### 2.1 Low total IgE

IgE is the principal mediator of type I hypersensitivity. As previously stated, the presence of IgE autoantibodies is a mechanism involved in the pathophysiology of CSU (26). Although specific IgE antibodies against classical common allergens such as aeroallergens or food allergens can be detected in some patients with CSU (in these cases the removal of the allergen could resolve the urticaria episode), these are not considered relevant to the development of the disease (26). However, more than 200 autoantigens recognized by IgE have been identified in patients with CSU, among which anti–IL-24 IgE has emerged as a common, specific, and functional autoantibody (27). In fact, treatment with omalizumab leads to a rapid neutralization of IgE antibodies, and the presence of specific IgE, such as anti–TPO IgE, may predict a faster therapeutic response in CSU patients (28).

Several studies have demonstrated that low total IgE levels are predictive of a reduced response to omalizumab (29–33). Fok et al. recently conducted a systematic review on predictors of treatment response in CSU patients and found that low serum total IgE was the only consistent predictor of poor or absent response to omalizumab (15).

In a retrospective multicenter observational study, Marzano et al. assessed serum total IgE levels in 340 patients with CSU treated with omalizumab (34). They found that IgE levels were 131.6 *±* 507 kUA/L in responders, significantly higher than in non-responders, whose levels were 42.1 ± 299 kUA/L (*p* < 0.0001). Nettis et al. also conducted a retrospective multicenter study to assess the efficacy and safety of omalizumab in 322 patients with CSU (35). They also assessed the predictive factors of response to treatment, categorizing pre-treatment IgE levels into quartiles. In a multivariate logistic regression model, IgE values between 48 and 236 kUA/L were less likely to be associated with poor response compared to IgE values under 48 kUA/L (*p* < 0.001). However, in the same study, IgE values > 236 kUA/L were not associated with a higher response rate to omalizumab (35). Another multicenter retrospective chart review involving 137 patients with CSU also demonstrated variable responses to omalizumab based on pre-treatment serum IgE quartiles (29). Patients were categorized into four quartiles according to their baseline serum IgE levels (1st quartile: 0-15.2 IU/mL, 2nd quartile: 15.3-68.8 IU/mL, 3rd quartile: 68.9-168.0 IU/mL, 4th quartile: 168.1-4,261 IU/mL). The multivariate logistic regression model revealed a statistically significant difference in treatment response across these quartiles (*p* < 0.001). Response rates were as follows: 48.4% in the 1st quartile, 86.1% in the 2nd, 88.2% in the 3rd, and 94.1% in the 4th quartile. Although this study also points towards the role of serum IgE levels as a predictive factor for omalizumab, these findings suggest that even patients with serum IgE levels as low as 15 kUA/L may benefit from omalizumab therapy, as nearly 50% of such patients respond to treatment (29).

Importantly, lower cutoff values for serum IgE have not been tested or recommended as thresholds below which omalizumab would be ineffective. Therefore, while serum IgE levels can serve as a predictive marker for omalizumab response, they should not be used in isolation to exclude patients from this treatment.

Finally, not only pretreatment serum IgE levels have been associated with treatment response, but also levels of IgE after 4 weeks of treatment and, more importantly, the ratio between 4-week and baseline IgE (36). A prospective observational study of 83 patients with CSU treated with omalizumab showed that non-responders to omalizumab had markedly lower baseline total IgE levels (17.9 IU/mL, 17.0–55.0 IU/mL), with respect to partial responders (82.0 IU/mL, 46.2–126.5 IU/ml, *p* = 0.008), or complete responders (73.7 IU/mL, 19.5–153.8 IU/mL, *p* = 0.032) (36). In addition, total IgE levels at week 4 of treatment were also lower in non-responders (17.9 IU/mL, 17.4–86.2 IU/mL) than in partial and complete responders (partial: 298.0 IU/mL, 205.8-543.5 IU/mL, *p* < 0.001; complete responders: 290.5 IU/mL, 121.5-637.5 IU/mL, *p* < 0.001), and increases in total IgE levels following omalizumab treatment were less pronounced in non-responders compared to responders. This study identified the ratio of total IgE at week 4 to baseline total IgE as the most reliable predictor of treatment response. Specifically, a lower increase in total IgE at week 4 correlated with poorer disease control. As it seems that the formation of complexes between IgE and omalizumab may contribute to the observed increase in total IgE levels in treated patients, the authors hypothesize that the diminished IgE elevation in non-responders may result from insufficient binding of IgE to the drug (36). However, further research is necessary to confirm these findings.

What appears to be clear is that measuring total IgE, an accessible and cost-effective technique, is advisable before initiating omalizumab treatment, as it is a useful tool in predicting the treatment response.

Key messages:

Lower pretreatment levels of serum IgE have been associated with poor treatment response.

A low ratio 4-week/baseline IgE may predict worse treatment outcomes.

No validated cutoff IgE levels exist to predict treatment response.

Patients with low IgE levels may also benefit from omalizumab treatment.

### 2.2 Type IIb autoimmune urticaria

As previously stated, in a subgroup of patients with CSU, the pathophysiological mechanism underlying the condition is the presence of IgG autoantibodies against IgE and its receptor (Fc*ε*RI), which provoke mast cell degranulation and the development of symptoms (37).

The diagnostic criteria of this subtype of CSU are (1) positive *in vivo* autoreactivity [a positive autologous serum skin test (ASST)] as evidence of serum factors capable of an inflammatory wheal response; (2) positive *in vitro* basophil reactivity [by basophil histamine release assay (BHRA) or basophil activation tests (BAT)] as evidence of serum factors causing histamine release, basophil activation or both; and, (3) a positive immunoassay for specific identification of IgG autoantibodies against IgE or Fc*ε*RI (western blot or ELISA) (38). Strictly meeting these criteria, less than 10% of patients are classified in this subtype of urticaria, and they typically exhibit high disease activity and are associated with other autoimmune diseases (37). Given that immunoassays for IgG autoantibodies and BAT are not always available, and the ASST is time-consuming and not always feasible in an outpatient setting, many authors have attempted to characterize and define biomarkers that could be used in routine clinical practice to more easily identify this subgroup of patients (7, 39).

A recent multinational, multicenter cross-sectional study of 182 patients with CSU assessed the demographic, clinical, and immunological profiles of patients with type IIb, or autoinmune, CSU (7). Although only 15 (8%) of the patients met the triple positivity criteria for this subgroup of urticaria (positive ASST, BAT, and IgG autoantibodies against IgE or Fc*ε*RI), they displayed a distinctive immunologic profile characterized by lower total IgE levels and higher IgG anti-TPO levels. Specifically, the IgE levels in patients with all three positive results were 22 (0–132) IU/mL, while they were 102 (23–1,401) IU/mL (*p* < 0.001) and 108 (2–909) IU/mL (*p* < 0.001) in patients with none or fewer than three positive results, respectively. In contrast, IgG anti-TPO levels were 153 (6–868) kU/L in patients with all three positive results, while they were 10 (0–211) kU/L (*p* < 0.01) and 9 (0–1,121) kU/L (*p* < 0.01) in patients with none or fewer than three positive results, respectively. Interestingly, the results of anti-TPO and IgE levels were similar in patients with all three positive tests as in those with positive *in vitro* basophil reactivity, which was 100% predictive of autoimmune CSU. In these patients, IgE and anti-TPO levels were inversely correlated (7). On the other hand, all clinical and demographic variables were similar across all CSU subgroups of patients, except for higher disease activity in patients with autoimmune CSU (*P* = 0.016) (7).

Basopenia has also been repeatedly associated with CSU type IIb or autoimmune forms (7, 39). Basophils are capable of releasing histamine and cytokines, including IL-4, IL-13, and IL-31, in response to the activation of IgE receptors on their surface or through IgE-independent pathways (40). Evidence suggests that a reduction in the number of basophils in peripheral blood, observed in patients with CSU, is associated with exacerbation of urticaria symptoms, likely due to the recruitment of these cells to lesional sites (40–42). Regarding eosinopenia, data from 1,613 patients with CSU were analyzed to clarify the role and relevance of eosinophil blood counts in CSU (24). The authors found that eosinopenia was associated with high disease activity, positive ASST and BHRA, low total IgE levels, and elevated CRP and IgG anti-TPO levels, suggesting that eosinopenia could also serve as a biomarker for type IIb CSU (24). Moreover, eosinophil levels were found to correlate with basophil levels (24)

In conclusion, there is a subgroup of patients with autoimmune or type IIb urticaria characterized by positivity for *in vitro* basophil reactivity, ASST, and IgG autoantibodies against IgE or IgE receptors. These patients can be identified in clinical practice by low IgE levels and high anti -TPO IgG levels, along with the presence of basopenia and eosinopenia. It has been suggested that such patients tend to exhibit more active CSU and a lower response to treatment with omalizumab (37, 43). However, this group of patients may show a good response to cyclosporine (37, 43). Thus, the recognition of these patients is particularly relevant in clinical practice. In line with these results, current European guidelines recommend measuring differential blood count, total IgE, and IgG anti-TPO antibodies as part of the routine initial workup in patients with CSU (2).

However, current evidence suggests that type IIb autoimmune CSU may serve as a biomarker for delayed therapeutic response to omalizumab (35, 44).

In a recent prospective study involving 64 patients with CSU treated with omalizumab, 39 patients (61%) demonstrated a rapid clinical response within 8 days (“early responders”), 17 patients (27%) responded between day 8 and 3 months (“late responders”), and 8 patients (12%) were classified as non-responders (44). Notably, patients with a positive BHRA exhibited a median time to response of 29 days, compared to a median of 2 days in BHRA-negative patients. Furthermore, only 1 of the 39 early responders was BHRA-positive, while 8 of the 17 late responders tested positive for BHRA (*P* = 0.0001) (44). A similar trend was observed with ASST positivity, which was significantly more prevalent among late responders than early responders (44). These observations were corroborated by a multicenter study evaluating the effectiveness of omalizumab in a larger cohort of 322 patients with CSU (35).

The authors postulated that in patients with type IIb autoimmune CSU, symptoms are mediated by IgG autoantibodies targeting either IgE or the high-affinity IgE receptor on the surface of mast cells, leading to their activation and degranulation (44). Omalizumab initially binds to and neutralizes circulating free IgE, which transiently increases the availability of unoccupied Fc*ε*RI receptors on mast cells. Over subsequent weeks, however, a downregulation of Fc*ε*RI expression occurs, ultimately reducing mast cell activation potential (44). Consequently, patients with a type IIb autoimmune CSU may experience a delayed clinical response to omalizumab due to this sequential immunological modulation (44). Nonetheless, further studies involving larger patient cohorts are warranted to confirm and refine these findings.

Key messages:

Patients with type IIb autoimmune urticaria may be identified in clinical practice by low IgE levels and high anti-TPO IgG levels, along with the presence of basopenia and eosinopenia.

Type IIb autoimmune CSU may exhibit a delayed clinical response to omalizumab.

### 2.3 Chronic inducible urticaria

CIndU is characterized by the appearance of wheals, and/or angioedema in response to specific triggers (45). Concomitant CIndU occurs in 7% to 30% of adult CSU patients (46). The most common type of CIndU is symptomatic dermographism followed by cold urticaria and delayed pressure urticaria (46). Mast cell degranulation plays a crucial role in the pathophysiology of CIndU, although the exact mechanism by which triggers induce this response remains unknown (47, 48). The diagnosis of CIndU relies on a thorough history and provocation testing according to the specific trigger suspected (48). It is crucial to identify the key clinical questions that raise suspicion for each CIndU subtype (47, 48). In summary, a directed clinical history should ask if symptoms such as pruritus or wheals appear in areas subject to scratching; after contact with water, cold or hot objects, or vibrations; in regions where clothing exert pressure or carry loads; following exercise or sweating; or after exposure to sunlight.

The treatment of CIndU includes the avoidance of specific and well-defined triggers (2). However, this strategy rarely achieves complete symptom control and is often associated with a significant impairment in quality of life, as strict avoidance of certain triggers is difficult to achieve and substantially interferes with daily activities (2). Moreover, patients with CSU and concomitant CIndU follow the same therapeutic algorithm as those with isolated CSU, with omalizumab being recommended when there is no response to antihistamines at fourfold doses (2).

Although concomitant CIndU has not been clearly associated with a poor response to omalizumab, its presence has been consistently linked to a longer duration of CSU (49, 50). A prospective observational study involving 142 patients with chronic urticaria—31% of whom had CIndU—was conducted to evaluate the determinants of omalizumab drug survival in real-world clinical practice (51). The authors reported overall omalizumab drug survival rates of 77%, 61%, and 55% at 1, 2, and 3 years, respectively (51). Treatment discontinuation was mainly due to well-controlled disease activity. In patients who discontinued omalizumab due to symptom control, multivariate Cox regression analysis identified the presence of CIndU as an independent predictor of longer drug survival, suggesting that patients with CIndU may represent a subgroup with a slower therapeutic response (51).

Similarly, a recent large multicenter cohort study of 2,325 patients with chronic urticaria initiating omalizumab treatment between 2009 and 2022 including 1,552 patients (67%) with CSU only, 595 (26%) with both CSU and CIndU, and 179 (8%) with CIndU only was conducted (52). The median omalizumab survival time was 3.3 years (95% CI, 2.9–4.0), primarily driven by disease control, and exceeded 5 years in patients predominantly affected by CIndU (52). Authors also observed that the presence of CIndU was not associated with a shorter time to discontinuation due to ineffectiveness, suggesting that omalizumab is effective in patients with CIndU, despite the need for longer treatment, possibly reflecting longer disease duration than in CSU (52). In fact, a prior prospective cohort study comparing 438 patients with isolated CSU with 111 patients with CSU and CIndU found that the presence of CIndU was associated with worse prognosis (17). Precisely, patients with CSU and CIndU required more frequent therapy after 5 years of follow-up than patients with isolated CSU (19.8% vs. 15.1%; *P* < 0.05), and presented significantly higher Urticaria Activity Score (UAS) scores (the sum of the UAS of the previous 3 weeks was 53.9 ± 28.0 in CSU with CIndU, while it was 33.5 ± 35.1 in patients with only CSU; *p* = 0.0005) (17).

In conclusion, although the presence of CIndU does not appear to be associated with a poorer response to omalizumab, it is linked to a less favorable disease prognosis and a greater need for prolonged omalizumab treatment. Therefore, identifying the presence of CIndU at the time of CSU diagnosis is advisable (48). Moreover, the presence of CIndU can often be suspected through a detailed medical history, which can be effectively obtained during an initial clinical visit.

Key messages:

Patients with CIndU may represent a subgroup with longer disease duration and a slower therapeutic response.

### 2.4 Obesity

Obesity is defined as a body mass index (BMI) ≥ 30 (53), and it results from complex interactions among genetic, socioeconomic, and cultural factors (54). Its rising prevalence is emerging as a significant global health concern in both adults and children, as it has been consistently associated with a high burden of comorbidities, including metabolic syndrome, cardiovascular diseases, neoplasms, and increased mortality (54). A cross-sectional study of 131 patients with chronic urticaria showed that 69 (52.7%) patients presented with central obesity, while it was present in 499 out of 1,285 age- and gender-matched controls (38.8%) (*p* = 0.002) (55). Similarly, 39 (29.8%) patients had metabolic syndrome compared to 17.8% in the control group (*p* = 0.001) (55). It was also observed that the presence of metabolic syndrome was a significant predictor for higher disease activity and lower rate of remission after antihistamines (55). The authors argue that both chronic urticaria and metabolic syndrome share a systemic pro-inflammatory state responsible for progression of symptoms (55). A recent large epidemiologic study involving 11,261 patients with CU and 67,216 controls also showed a significant association between chronic urticaria and metabolic syndrome (OR = 1.12, 95% CI 1.1–1.2, *P* < 0.001) and obesity (OR = 1.2, 95% CI 1.1–1.3, *P* < 0.001) (56).

Magen et al. conducted a retrospective observational study involving 106 patients treated with omalizumab for CSU, analyzing the impact of comorbidities on treatment response (31). They reported that 37.5% of non-responders were obese, compared to only 12.7% of complete responders (*p* = 0.02) (31). In multivariate analysis, obesity was associated with non-response to omalizumab (odds ratio 3.42, 95% CI 2.04–5.17, *p* < 0.001) (31). The authors hypothesized that adipokines and proinflammatory cytokines, which are elevated in obesity and metabolic syndrome, may directly activate mast cells, thereby worsening CSU severity and influencing treatment response (31).

However, it has been suggested that obese patients may not respond to the standard licensed dose of omalizumab (300 mg every four weeks) but may benefit from dose escalation (14). To evaluate the effectiveness of omalizumab dose escalation in patients with CSU refractory to the licensed dose, the medical records of 286 patients treated with omalizumab were retrospectively reviewed (14). The authors found that 65% of patients achieved good disease control (UAS7 ≤ 6) with the standard dose, and 75% of partial or non-responders responded following dose escalation (14). The only pretreatment variables that significantly differed between responders to the standard dose and those who required higher doses were age (OR 1.038, *p* = 0.013) and BMI (OR 1.14, *p* = 0.004), suggesting that obesity may predict non-response to the licensed dose, but not to an increased dose (14).

In other dermatological conditions such as psoriasis, the clinical efficacy of biologic therapies decreases as body mass index increases (57), possibly due to greater volume of distribution and clearance in individuals with higher body weight (58). Consequently, weight-based dose adjustments are recommended for some biologics. However, the volume of distribution of omalizumab approximates the plasma volume (59), and population pharmacokinetic analyses suggest that dose adjustments are not required based on age (6–76 years), race, ethnicity, gender, or BMI (60). Nevertheless, in asthma treatment, omalizumab dosing is adjusted based on BMI and IgE levels (61).

Although there is no formal requirement to adjust the initial omalizumab dose for CSU based on body weight, in clinical practice—and as previously discussed—omalizumab dose escalation should be considered in partial or non-responders, particularly when BMI is high, as the likelihood of treatment success may increase under these conditions (14).

Key messages:

Obesity and metabolic syndrome have been associated with the presence of urticaria.

Obese patients may be refractory to the licensed dose of Omalizumab, but might benefit from dose escalation.

## 3 Predictors of recurrence of chronic spontaneous urticaria

CSU is recognized as a relapsing disease with variable duration. While symptoms often improve over time (62), there are few studies assessing its natural history and they have yielded conflicting results regarding disease duration (62, 63). A recent cross-sectional, single-center study involving 72 patients with CSU found that 56 patients (78%) experienced resolution of their condition (63). The median duration of urticaria among these patients was 48 months, with a range from 2 to 204 months (63). In this cohort, 30% of patients experienced symptom duration between 6 and 23 months, 16% between 24 and 47 months, 20% between 48 and 71 months, 9% between 72 and 95 months, another 9% between 96 and 119 months, and 16% had symptoms persisting for more than 120 months (63). The same study reported that 22 out of 72 patients (31%) experienced a recurrence of their disease following initial remission, with a mean remission duration of 21 ± 10 months (63). A Phase 3b, randomized, multicenter, open-label clinical trial evaluating omalizumab at 150 mg or 300 mg as initial therapy or following treatment withdrawal and symptom relapse in 314 patients with CSU demonstrated that 56 of 115 omalizumab responders experienced symptom relapse after an 8-week period of withdrawal, representing a relapse rate as high as 49% (64). Another multicenter observational retrospective study involving 470 CSU patients showed that 236 out of 392 responders to treatment presented with a relapse, which means a relapse rate of 60.2% (34)

Therefore, symptom relapse following omalizumab discontinuation may represent a common scenario in clinical practice. Several studies have investigated potential predictive factors for CSU relapse following omalizumab discontinuation, yielding variable results (34, 65–69). Identifying such predictors is crucial for optimizing patient management. A better understanding of these factors allows clinicians to personalize treatment, set realistic expectations, and potentially enhance overall therapeutic success.

### 3.1 Omalizumab optimization

The concept of omalizumab optimization refers to the gradual tapering of the drug prior to treatment withdrawal (69). Different strategies have been proposed to initiate this process: either reducing the dose to 150 mg every 4 weeks (70), or extending the administration interval while maintaining the same dose (71). A recent retrospective, real-world, multicenter study involving 257 patients with CSU whose therapy with omalizumab was optimized showed that both strategies were comparable in terms of disease control (72). The authors hypothesized that dose reduction might be a more cost-effective approach to optimization (72, 73).

Most studies evaluating omalizumab recurrence do not specify the withdrawal method, suggesting that no optimization strategy was employed (34, 65, 67). Recently, a prospective multicenter study including 131 patients with CSU who discontinued omalizumab after optimization—regardless of the optimization method used—and with a minimum follow-up of 12 months was conducted (69). The authors reported a relapse rate of 32.8% at a median time of 3.00 months (IQR, 2.00–4.50) (69). When compared with rates in the literature, which may reach up to 60%, this finding suggests that omalizumab optimization may reduce recurrence after discontinuation and could represent a viable strategy for this purpose (34, 69). Nevertheless, other authors have reported recurrence rates of approximately 30% following omalizumab discontinuation without specifying whether optimization was applied (63). To date, no studies have directly compared relapse rates between patients who did or did not undergo optimization, so considering optimization as a predictor of lower recurrence should be approached with caution.

An important point to consider is that the optimization strategy should ideally be initiated when patients achieve complete disease control. Further treatment modifications should also be made only when disease control is maintained (70). In this regard, Ceravalls et al. showed that achieving a complete response before omalizumab optimization was significantly associated with a lower relapse rate: 95.5% of patients who did not relapse after omalizumab discontinuation had achieved a complete response, compared to 83% of those who relapsed (*P* = 0.023) (72).

Another retrospective observational study evaluating omalizumab use in clinical practice in 134 patients analyzed treatment outcomes across various dosing patterns, including licensed, reduced, and increased doses (74). In the same line, the authors found that among patients who relapsed after omalizumab discontinuation, the median time to relapse was 5 months, regardless of the number of doses received at the licensed dose (74). This suggests that once disease control is achieved, prolonged treatment at the initial dose may not contribute to long-term remission (74).

Current evidence suggests that responses to omalizumab reintroduction after discontinuation are maintained and comparable to initial responses (75, 76). In the series by Ceravalls et al., omalizumab was restarted in 81.3% of patients, and 34.28% were reinitiated with an optimized dosage, achieving complete response (69). Similarly, in the series by Akdas et al., re-treated patients who resumed the same optimized dose discontinued previously responded similarly to their initial treatment (74). Thus, patients who relapsed after optimization may benefit from reintroducing the last effective tapered dose instead of reverting to 300 mg every 4 weeks (69, 70).

To sum up, omalizumab optimization—tapering the dose either by reducing it or gradually extending the dosing interval once symptoms are well controlled—followed by reintroduction at the minimum effective dose upon relapse, may be a promising strategy to reduce treatment burden. This approach may enable prolonged treatment in patients who require it, representing a more cost-effective option. However, further studies—ideally prospective and controlled—are needed to clarify the role of this strategy in reducing recurrence.

Key messages:

Omalizumab optimization refers to the gradual tapering of the drug prior to treatment withdrawal.

Omalizumab optimization initiated after complete disease control may be associated with lower relapse rate.

### 3.2 Duration of symptoms

A longer duration of CSU symptoms is one of the most frequently reported factors associated with an increased likelihood and faster onset of relapse following omalizumab discontinuation (34, 63, 69, 72, 77). In a retrospective multicenter study involving 470 CSU patients, Marzano et al. observed that the duration of disease was significantly longer in patients who experienced either a first relapse (median duration: 24 vs. 13 months, *P* < 0.0001) or a second relapse (median duration: 25 vs. 18 months, *P* = 0.0105) after discontinuation of omalizumab following the first, or the second treatment course, respectively (34).

Furthermore, a cross-sectional study involving 72 CSU patients receiving omalizumab showed that among the 56 patients in remission at the time of the study, 5 out of 15 patients (33%) who had experienced a relapse had a disease duration longer than 10 years. In contrast, only 4 out of 41 patients (9.8%) who did not report a relapse had a disease duration exceeding 10 years (*P* = 0.033) (63).

A topic of interest is whether predictive factors for recurrence change in patients who have discontinued treatment following optimization. In their retrospective study of 131 CSU patients treated with omalizumab, Ceravalls et al. found that disease duration remained a predictive factor in this context (69). According to their findings, the median disease duration in patients who did not experience relapse was 11 months (interquartile range: 6–62), compared to 24 months (10.5–95.75) in those who did relapse (*p* = 0.032) (69).

Furthermore, the same group conducted a retrospective analysis in the same CSU cohort who discontinued omalizumab after optimization, with a minimum follow-up of six months, comprising 257 patients, to compare outcomes across different optimization strategies (72). They observed that, irrespective of the optimization method, recurrence was associated with a longer disease duration (21 months in relapsing patients vs. 13 months in non-relapsing patients, *p* = 0.0148) (72).

In the skin affected by CSU, there is a sustained perivascular infiltrate of T cells, basophils, along with vasodilation, reflecting persistent inflammation (78). The longer the disease duration, the more this inflammatory state is maintained, since the presence of pro-inflammatory mediators promotes immunological adaptation mechanisms that stabilize mast cell activation and reinforce the chronic response (5), thereby facilitating symptom recurrence after drug withdrawal.

Nevertheless, some studies specifically investigating predictors of relapse have not confirmed a consistent association between disease duration and the risk of recurrence (15, 68, 76). Further research is warranted to elucidate the relationship between disease duration and post-discontinuation outcomes in patients treated with omalizumab.

Key messages:

A longer disease duration has been associated with a higher recurrence rate, possibly due to a maintained inflammatory state promoting persistent mast cell activation.

### 3.3 Disease activity

Most authors assess disease activity—understood as the intensity of urticaria symptoms—using the UAS (79), in accordance with current European guideline recommendations (2). The UAS7 captures the severity of pruritus and the number of hives over the previous seven days, with scores ranging from 0 (no symptoms) to 42 (maximum disease activity) (68). This tool has demonstrated good correlation with patient-reported symptom diaries as well as with health-related quality of life (79).

Ferrer et al. conducted a pooled analysis of data from two randomized controlled trials in patients with CSU—ASTERIA I (80)(*n* = 319; omalizumab 75, 150, or 300 mg or placebo every 4 weeks) and ASTERIA II (81) (*n* = 323; same arms of dosing regimen)—to develop a regression algorithm identifying the strongest predictors of rapid symptom recurrence following omalizumab discontinuation (68). They found that lower baseline UAS7 scores and a rapid treatment response—quantified by a high UAS7 area above the curve calculated from UAS7 values between weeks 0 and 4—were associated with a lower probability of rapid symptom return (68). In contrast, other variables, including the presence of angioedema, age, sex, body weight, duration of CSU, itch severity score, omalizumab dose, number of doses administered, CU Index IgE levels, and pre- or post-baseline medications, did not predict symptom recurrence (68).

Marzano et al. also reported that, in multivariate analysis, a higher baseline UAS7 score, along with longer disease duration, was significantly associated with an increased risk of first relapse following omalizumab discontinuation (*P* = 0.0029) (34). In another retrospective, single-center study, 30 out of 100 patients with CSU who discontinued omalizumab experienced relapse. Multivariate Cox regression analysis revealed that a high UAS7 after 24 weeks of treatment was significantly associated with a higher risk of relapse, with each additional point increasing the risk by 5.4% (82)

In the same line, Ceravalls et al. also observed that patients who experienced relapse after omalizumab optimization also required more frequent up-dosing (30% vs. 15%, *P* = .0038) and had a lower complete response percentage before optimization (84.64% vs. 94.75%, *P* = .0075) compared with patients who did not relapse (72). These findings suggest that patients with more difficult-to-control CSU are more likely to experience relapse (72).

It has been previously suggested that certain features are typical of type IIb CSU, with higher disease severity being one of them (37). Similarly, elevated CRP levels have been associated with increased urticaria activity (83). However, this finding has not been consistently confirmed in the literature (84, 85). Nevertheless, it has been proposed that elevated CRP may be related to an autoimmune mechanism underlying CSU (2, 86), in parallel with decreased total IgE levels, elevated anti-TPO IgG titers, basopenia, and eosinopenia (37). Large-scale prospective studies are needed to confirm these potential predictive biomarkers and to identify new ones in order to advance personalized medicine in CSU.

Key messages:

Higher disease activity, assessed by higher scores in the UAS7 questionnaire or by the need of omalizumab up-dosing, may predict a higher risk of relapse.

## 4 Discussion

This narrative review aims to summarize the evidence regarding predictors of response to omalizumab (Table 1) and the risk of relapse following its discontinuation (Table 2), with a particular focus on variables that are accessible in most clinical settings. The objective is to provide a practical and applicable guide for physicians involved in the management of CSU across diverse healthcare environments. This review is inherently limited by its methodology, as it does not follow the systematic processes designed to minimize bias. In particular, the selection of articles may be subject to selection bias, and the scope of included evidence is narrower than in systematic reviews. However, the main aim of this review is to focus on aspects more directly related to real-world practice and applicable to clinical care; therefore, the search strategy was oriented towards such studies.

**Table 1:** Summary of main predictors of response to omalizumab.

Predictors	Study (year)	Study design (n)	Results	Conclusion
Low total IgE	Marzano et al. (2019)	Retrospective observational study (340)	IgE levels were 131.6 kUA/L in responders vs. 42.1 kUA/L in non-responders; *p* < 0.0001.	Lower pretreatment levels of serum IgE have been associated with poor treatment response.
	Nettis et al. (2018)	Retrospective observational study (322)	IgE values under 48kUA/l more likely to be associated with poor response; *p* < 0.001.	
	Straesser et al. (2018)	Retrospective chart review (137)	48.4% of patients with serum IgE levels of 0-15.2 IU/mL responded vs. 86.1% with IgE levels of 15.3–68.8 and 88.2% with IgE levels of 68.9–168.0; *p* < 0.001.	
	Ertas et al. (2018)	Prospective observational study (83)	Increases in total IgE levels following omalizumab treatment were less pronounced in non-responders; *p* = 0.032.	A low ratio 4-week/baseline IgE may predict worse treatment outcomes.
Type IIb autoimmunity	Gericke et al. (2017)	Prospective observational study (64)	1/39 early responders was BHRA-positive, while 8/17 late responders tested positive for BHRA; *p* = 0.0001	Patients with type IIb autoimmune CSU may experience a delayed clinical response to Omalizumab.
	Nettis et al. (2018)	Retrospective observational study (322)	ASST positivity was more prevalent among late responders; *p* < 0.001.	
Chronic inducible urticaria	Spekhorst et al. (2019)	Prospective observational study (142)	CIndU was identified as an independent predictor of longer drug survival.	Patients with CIndU may represent a subgroup with a slower therapeutic response.
	Soegiharto et al. (2024)	Cohort study (2325)	Omalizumab survival time was 3.3 years (95% CI, 2.9–4.0), while it exceeded 5 years in patients affected by CIndU.	
	Curto-Barredo et al. (2018)	Prospective cohort study (438)	CIndU required more frequent therapy after 5 years of follow-up than patients with CSU (19.8% vs. 15.1%); *p* < 0.05.	
Obesity	Magen et al. (2019)	Retrospective observational study (106)	Obesity was associated with poor response to omalizumab; Odds ratio 3.42, 95% CI 2.04–5.17; *p* < 0.001.	Obese patients may be refractory to the licensed dose of Omalizumab, but might benefit from dose escalation.
	Curto-Barredo et al. (2018)	Retrospective observational study (286)	BMI differed between responders to the licensed dose and those who needed an increased dose; *p* = 0.004.	

Biomarkers and clinical factors associated with delayed and non-response to Omalizumab in patients with Chronic Spontaneous Urticaria (CSU).

BHRA, basophil histamine release assay; ASST, autologous serum skin test; BMI, body mass index.

**Table 2:** Summary of main predictors of relapse after omalizumab discontinuation.

Predictors	Study (year)	Study design (n)	Results	Conclusion
Omalizumab optimization	Ceravalls et al. (2025)	Retrospective observational study (131)	Relapse rate in patients who discontinued after optimization was found of 32.8% and occurred at a median time of 3.00 months (IQR, 2.00–4.50).	Omalizumab optimization after complete disease control may be associated with lower relapse rate.
	Ceravalls et al. (2025)	Retrospective observational study (257)	95.5% of patients who did not relapse after discontinuation had achieved a complete response vs. 83% of those who relapsed; *p* = 0.023	
Long duration of symptoms	Marzano et al. (2019)	Retrospective observational study (470)	Longer duration of disease was found in patients who relapsed: 24 vs. 13 months in those who did not; *p* = 0.0105	Long duration of disease seems to predict higher recurrence rate.
	Stepaniuk et al. (2020)	Prospective observational study (72)	33% of patients who relapsed had a disease duration >10 years vs. 9.8% of those who did not relapse; *p* = 0.033	
	Ceravalls et al. (2025)	Retrospective observational study (131)	Median disease duration in patients who did not relapse was 11 months vs. 24 months in those who did relapse; *p* = 0.032	
	Ceravalls et al. (2025)	Retrospective observational study (257)	Disease duration was of 21 months in relapsing patients vs. 13 months in non-relapsing ones; *p* = 0.0148	
Disease activity	Ferrer et al. (2018)	Pooled analysis from 2 randomized controlled trials (642)	A lower baseline UAS7 and a rapid treatment response was associated with a reduced likelihood of rapid symptom return.	Lower baseline UAS7 is associated with lower risk of relapse.
	Marzano et al. (2019)	Retrospective observational study (470)	A higher baseline UAS7 was associated with increased risk of relapse; *p* = 0.0029	
	Kucharczyk et al. (2025)	Retrospective cohort study (100)	Each additional point in UAS7 increased the risk of recurrence by 5.4%.	

Biomarkers and clinical factors associated with recurrence after Omalizumab discontinuation in patients with Chronic Spontaneous Urticaria (CSU).

IQR, interquartile range; UAS7, urticaria activity score.

The predominant underlying pathophysiological mechanism of CSU appears to be relevant in predicting treatment response. As previously described, CSU can be broadly categorized into type I autoimmune (autoallergic), mediated by IgE autoantibodies, and type IIB autoimmune, mediated by IgG autoantibodies directed against IgE or its high-affinity receptor Fc*ε*RI (37)**.**

Regarding response to omalizumab, patients with a type IIB autoimmune profile may exhibit a poorer therapeutic response. These patients are characterized by positive *in vitro* BAT, positive ASST, and the presence of IgG autoantibodies against IgE or Fc*ε*RI. These patients can be detected in clinical practice by additional features commonly observed in this subgroup such as low total IgE levels, elevated anti-TPO IgG titers, and analytical or clinical markers such as basopenia, eosinopenia, elevated CRP, and greater disease activity (37).

The presence of CIndU is associated with the need for longer duration of omalizumab therapy (51). Obesity may also impact treatment outcomes, with some patients failing to respond to standard doses but responding to higher doses (14). While current guidelines do not require weight-based dosing, dose escalation should be considered in partial or non-responders, as some of these patients, particularly when BMI is high, may still benefit from higher doses (14).

Recognizing these patient profiles is particularly relevant in clinical practice, as it allows for more informed treatment decisions and realistic patient counseling. Following the current international CSU guidelines (2), the authors recommend implementing a structured checklist at the initial visit, including clinical variables (age, sex, weight, height), CSU-specific data (symptom duration, angioedema, previous flares, and associated CIndU), and assessment of disease activity using validated tools such as the UAS7 score. Initial laboratory tests should include CRP, ESR, total IgE, D-dimer, thyroid function, anti-TPO and anti-thyroglobulin antibodies, and a complete blood count including eosinophils and basophils. Most of these variables can be obtained through a basic clinical interview and routine laboratory tests.

Despite the presence of predictors of poor response, omalizumab remains the recommended next-line treatment after failure of up-dosed antihistamines (2), as a significant proportion of patients with poor prognostic factors, such as low IgE, still respond to treatment (29). Although omalizumab is an anti-IgE monoclonal antibody, its mechanism involves reducing free IgE levels, leading to downregulation of Fc*ε*RI on mast cells and basophils and thereby limiting their capacity to respond to allergen exposure and their further activation (87). Indeed, a study in 47 patients with CSU showed an 88.1% reduction in basophil Fc*ε*RI expression four weeks after omalizumab administration (32).

It has been proposed that omalizumab may act through distinct mechanisms in different patient subgroups—rapid responders may benefit from direct IgE blockade, while slow or non-responders may improve via gradual downregulation of Fc*ε*RI (44). Consequently, it is the authors’ opinion that continuing omalizumab therapy is advisable even in the absence of an early clinical response, including in cases where escalation to additional therapies such as cyclosporine is considered.

Importantly, some predictors of poor response to omalizumab have been associated with favorable response to cyclosporine (43, 88). For example, in an analysis of 398 CSU patients who underwent a BHRA, 81% of those with a positive result achieved complete remission with cyclosporine, compared to only 19% with a negative result (*P* < 0.0001) (89).

Cyclosporine is a calcineurin inhibitor that acts by inhibiting the release of inflammatory cytokines from T cells, such as IL-2, IL-3, IL-4, and TNF, thereby reducing mast cell activation and degranulation (90). Accordingly, it is reasonable to assume that cyclosporine exerts its greatest effect in type IIB autoimmune mechanisms (88).

Following this rationale, a possible strategy for patients who do not achieve adequate disease control with omalizumab could be the addition of cyclosporine. Several case series have demonstrated the efficacy and safety of low-dose CsA in combination with omalizumab in omalizumab non-responders (91–93).

Regarding relapse prediction, some relevant factors are likely the duration of urticaria and disease activity (34, 72). Higher urticaria activity has classically been considered part of type IIB autoimmunity (37). Accordingly, some studies have shown that other features of this type of immunity, such as IgE levels or anti-TPO antibodies, may also be associated with relapse risk (72) Nevertheless, there are few studies addressing this issue, with variable results; therefore, further research is needed. However, again, the pathophysiological mechanism underlying urticaria appears to play a crucial role not only in treatment response but also in the likelihood of recurrence following therapy.

Finally, a progressive reduction of omalizumab treatment—known as treatment optimization—has been proposed as a potential predictor of a lower probability of relapse after omalizumab discontinuation (69). Moreover, current evidence suggests that patients who achieve a complete response prior to treatment discontinuation may have a lower risk of recurrence (72). Thus, in clinical practice, in order to minimize the risk of recurrence, it may be advisable to continue omalizumab treatment until complete remission is achieved, followed by an optimization strategy consisting of gradual dose de-escalation prior to discontinuation (70).
